# Primary Biliary Tract Cancers in Golestan, Iran: 13-Year Experience of Golestan Population-Based Cancer Registry

**DOI:** 10.34172/aim.2023.76

**Published:** 2023-09-01

**Authors:** Ali Ashkbari, Fazel Isapanah Amlashi, Sima Besharat, Mostafa Mofidi, Taghi Amiriani, Abdolreza Fazel, Mehdi Alimadadi, Faezeh Salamat, Seyyed Mehdi Sedaghat, Somayeh Livani, Ali Bagheri, Shahriyar Semnani, Alireza Norouzi, Gholamreza Roshandel

**Affiliations:** ^1^Golestan Research Center of Gastroenterology and Hepatology, Golestan University of Medical Sciences, Gorgan, Iran; ^2^Cancer Research Center, Golestan University of Medical Sciences, Gorgan, Iran; ^3^Omid Cancer Research Center, Golestan University of Medical Sciences, Gorgan, Iran; ^4^Deputy of Public Health, Golestan University of Medical Sciences, Gorgan, Iran

**Keywords:** Biliary tract cancer, Epidemiology, Gallbladder cancer, Gastrointestinal cancer, Iran

## Abstract

**Background:**

Epidemiological research on the high-risk population might be helpful in early detection and prevention of biliary tract malignancies. This study assesses the prevalence of biliary tract cancer (BTC) in the Golestan province, northeastern Iran, between 2004 and 2016.

**Methods:**

The current study used information from the Golestan Population-based Cancer Registry (GPCR) to access the epidemiology of BTC across a 13-year period while taking into account temporal and geographic differences. The number of cases, crude rates, age-standardized incidence rates (ASRs) per 100,000 person-years, average annual percent change (AAPC), age-specific incidence rates, and 95% confidence intervals (CI) were reported for each year with respect to gender and place of residence.

**Results:**

Totally, 224 instances of BTC overall (54% of whom were females) were reported throughout the research period. The ASR of BTC was 1.7 (95% CI: 1.4‒2) for females and 1.4 (95% CI: 1.1‒1.6) for men, respectively. Males exhibited a growing time trend in incidence (AAPC: 7.18; CI: 0.06‒14.81; *P*-value:0.048), whereas females had a decreasing trend (AAPC: 0.82; CI: -5.94‒4.57; *P*-value: 0.740). Both sexes saw an increase in age-specific incidence rates starting at the age of 45; however, males experienced a significant increase in incidence in the age group of 75 to 79 while the female rates grew steadily.

**Conclusion:**

The focus for cancer control in this region may be given to demographic groups with a combination of risk factors, including male gender, older age, and urban residence.

## Introduction

 More than 70% of the incidence of biliary tract cancers (BTC) and their related deaths occur in Asia, and Iran is among the low-risk regions in this continent.^1^ The concern is that the global cancer observatory (GCO) estimated BTC incidence and mortality rates will rise two times by 2040 in Iran.^1^ However, there is still lack of details and analysis about the current state of BTC in Iran. Thus, investigating the population at higher risk for BTC, as well as its temporal and spatial features, is necessary for clinical and public health management.

 BTCs are uncommon cancers with poor prognosis and low survival rates, generally diagnosed at advanced stages. They are sub-classified as intrahepatic cholangiocarcinoma (iCCA), extrahepatic cholangiocarcinoma (eCCA), gallbladder cancer (GBC), and ampulla of Vater cancer (AVC).^2,3^ BTCs account for less than 1% of the world’s cancers, with around 115,949 new cases and 84,695 related deaths.^4^ In 2020, Chile, China, India, Japan, and the Republic of Korea showed the highest incidence rates worldwide, while the rate was lower among Western countries.^5,6^ In Iran, BTC constituted around 0.5% of new cases of cancer in 2020. Evidence showed that between 2000 and 2016, the incidence of BTC had a declining trend in Iran,^7^ while its mortality rate increased from 1990 to 2015.^8^ The increasing trend of mortality rate could be associated with a high prevalence of diabetes and obesity, improvement in diagnosis, and inadequate access to cholecystectomy surgeries.^8^ Similar to the global rate, the incidence and mortality rates of BTC were higher among females in Iran. This gender difference could be related to some risk factors such as a history of gallstones, pregnancy, labor, and oral contraceptive pill (OCP), making females prone to higher risk of BTC. Nevertheless, the risk factors of BTC have not been deeply evaluated in Iran and need further investigations.

 The Golestan Population-based Cancer Registry (GPCR) was established in 2004 in the Golestan province, northeastern Iran, to provide comprehensive statistics for cancer control in the high-risk population of the region, which is located in the Asian belt of upper-gastrointestinal cancers.^9^ The main objective of the present study, based on GPCR resources, is to report the trends and incidence rate of BTC by considering temporal and spatial variations from 2004 to 2016 in the Golestan province.

## Materials and Methods

###  Study Design

 The present study included all the data about BTC cases in the Golestan province from 2004 to 2016. Golestan is located in northeastern Iran with a total population of 1 868 819 (men: 50.21%) according to the 2016 census by the statistical center of Iran.^10^

###  Source of Data

 All the data on BTC cases were obtained from the GPCR.^9^ The GPCR was established in the Golestan province in 2004, and it has been a voting member of the International Agency for Research on Cancer (IARC) for around 15 years.^11^ Since 2004, GPCR has collected data on all cancer cases in Golestan and neighboring provinces such as Khorasan Razavi, Mazandaran, and Tehran. The GPCR process of registration includes primary cancers based on the definitions and protocols developed by IARC and the International Association of Cancer Registries (IACR).

###  Data Collection

 The GPCR establishment has already provided a detailed explanation of the cancer registry approach.^9^ In brief, GPCR receives data from the deputy of treatment affairs of the GOUMS. All the potential sources of data are considered in data collection, including all public and private diagnostic and therapeutic centers in this region (hospitals, pathology/laboratory centers, imaging centers, and selected specialist physician’s offices) and primary health centers. Also, GPCR receives data from centers in other provinces such as Tehran, Mazandaran and Razavi Khorasan to increase the reliability of the data. Data are collected both actively and passively by well-trained registry staff that regularly visit all centers. Also, information is collected about the cases who refer to healthcare centers outside the registration area.

 To maintain data quality, data quality indicators (including the ratio of microscopically verified cases (%)), cases diagnosed only by the death certificate, cases with unknown age, and cases with an unknown primary site) are calculated and controlled routinely.

###  Definitions

 Tumor features such as topography, morphology, behavior, and grade were classified based on the third edition of the International Classification of Disease for Oncology (ICD-O-3).^12^ The corresponding code for gallbladder cancers is C23.9 in ICD-O-3. Cancers in other and unspecified parts of the biliary tract, including extrahepatic bile duct, ampulla of Vater, overlapping lesion of the biliary tract, and biliary tract, and not otherwise specified (NOS) are coded C24.0, C24.1, C24.8 and C24.9, respectively.

###  Statistical Analysis

 GPCR used the CanReg5 software for data entry and analysis.^13^ The number of cases, crude rates, age-standardized incidence rates (ASRs), and 95% confidence intervals (CIs) of ASRs of BTC in Golestan were calculated for each year with respect to gender and place of residence using the Segi-Doll world population. All rates were presented per 100 000 person-years. Average annual percent change (AAPC) with 95%CI was calculated by log-linear join point regression to assess trends over time.^9^

## Results

 A total of 224 BTC cases (54% female) were registered from 2004 to 2016 in the Golestan province. The crude rates for females and males were 1.1 and 0.9, respectively. The total ASR of BTC for females was 1.7 (95% CI: 1.4‒2), and the highest rate was recorded in 2014 (ASR: 2.3). In males, the total ASR was 1.4 (95% CI: 1.1‒1.6), and the highest rate was 3.2 that occurred in 2014, the same as females. Overall, males showed higher ASR in the study’s last three years than females. The numbers of cases, crude rates, ASR, and 95% CI of ASRs of BTC by gender, residence area, and year are presented in Table 1.

**Table 1:** Incidence and Frequency of BTC in Golestan, Iran by Demographic Information

		**Male**				**Female**			
		**Number**	**Crude rate**	**ASR**	**95% CI of ASR**	**Number**	**Crude Rate**	**ASR**	**95% CI of ASR**
Residence	Total	103	0.9	1.4	1.1–1.6	121	1.1	1.7	1.4–2.0
	Urban	62	1.1	1.6	1.2–2.0	71	1.2	1.9	1.4–2.4
	Rural	41	0.7	1.1	0.8–1.5	50	0.9	1.4	1.0–1.8
Year	2004	4	0.5	0.8	0.0–1.7	11	1.4	2.3	0.9–3.7
	2005	4	0.5	1.0	0.0–1.9	10	1.2	1.9	0.7–3.1
	2006	9	1.1	1.8	0.5–3.0	8	1.0	1.8	0.4–3.2
	2007	4	0.5	0.8	-0.1–1.8	6	0.7	1.3	0.2–2.4
	2008	4	0.5	0.7	0.0–1.4	5	0.6	1.0	0.1–1.8
	2009	4	0.5	0.8	0.0–1.5	7	0.8	1.0	0.2–1.7
	2010	6	0.7	1.1	0.2–2.0	12	1.4	2.2	0.9–3.5
	2011	8	0.9	1.3	0.3–2.2	8	0.9	1.3	0.4–2.3
	2012	4	0.5	0.7	0.0–1.4	11	1.2	1.8	0.7–3.0
	2013	8	0.9	1.3	0.4–2.3	12	1.3	2.1	0.8–3.3
	2014	21	2.3	3.2	1.7–4.6	14	1.5	2.3	1.0–3.5
	2015	16	1.7	2.4	1.2–3.6	8	0.9	1.2	0.3–2.1
	2016	11	1.2	1.6	0.6–2.5	9	1.0	1.4	0.4–2.3

**Figure 1:**
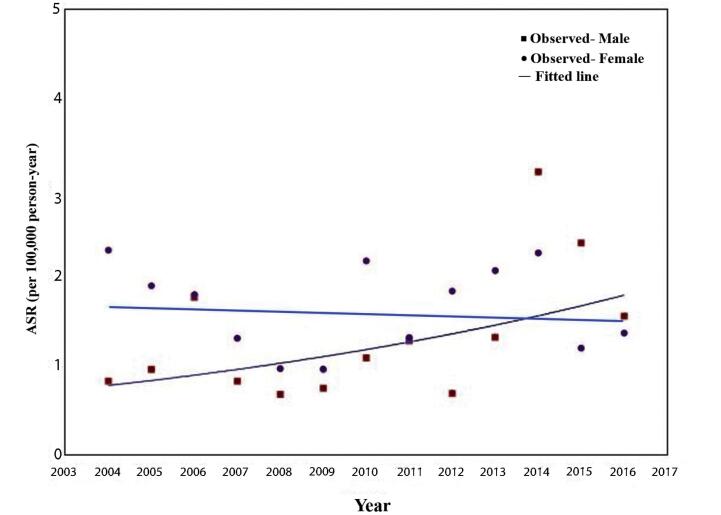
Temporal Variations in Age-Standardized Incidence Rate of BTC in Golestan, Iran, by gender (2004–2016)

 The time trend in incidence decreased slightly in females (AAPC: 7.18; CI: 0.06‒14.81) over the years without a significant change (*P*-value: 0.740). At the same time, males had a significantly increasing trend in incidence (AAPC: 7.18; CI: 0.06‒14.81; *P*-value: 0.048). The time trends for incidence and temporal variations in ASRs are presented in Table 2 and Figure 1.

**Table 2:** Time Trends in Incidence of Biliary Tract Cancers in Golestan, Iran (2004‒2016), by Gender

	**Time Period**	**AAPC**	**95% CI of AAPC**	***P*** Value^*^
Male	2004‒2016	7.18	0.06 to 14.81	0.048
Female	2004‒2016	-0.82	-5.94 to 4.57	0.740

AAPC, average annual percent change; CI, confidence interval. *Level of significancy: *P* value < 0.5

**Figure 2:**
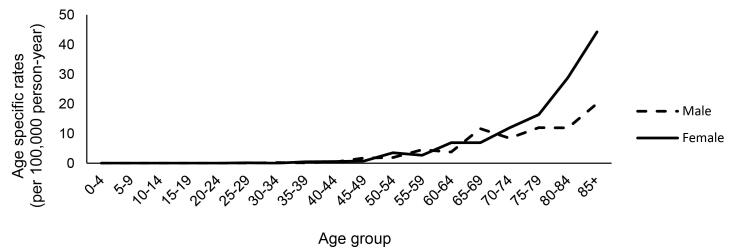
Age-Specific Incidence Rates (Per 100 000 Person-Year) of BTC in Golestan, Iran by Gender (2004–2016)

 The age-specific incidence rate began its upward slope at the age of 45 and continued to grow with age in both sexes (Figure 2). In males, the rate considerably accelerated at the age of 70‒74 and reached the peak in the age group 85 years and older. In females, the incidence constantly increased and did not show the same significant rise in the older age groups. Regarding ASR, males showed a higher rate in almost every age group older than 60.

**Figure 3:**
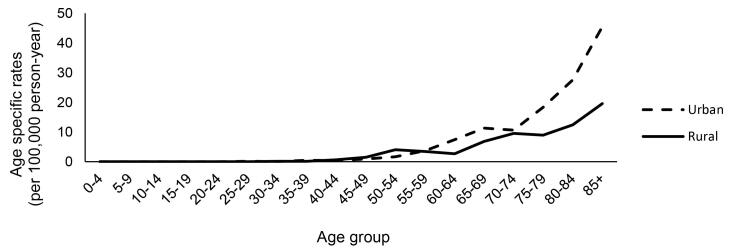
Age-Specific Incidence Rates (Per 100 000 Person-Year) of BTC in Golestan, Iran by Residence Area (2004–2016)

 The rates of BTC were higher in urban compared to rural areas (59.2 vs. 40.8) (Table 1). In urban cases, the ASR for females and males were 1.9 (95% CI: 1.4‒2.4) and 1.6 (95% CI: 1.2‒2.0), respectively, higher than the rural equivalents. As shown in Figure 3, the age-specific incidence rate started to increase at the age of 45 in both areas. The urban cases had higher ASR in all age groups over 60. In the age group 70-74, there was a significant rise in the incidence of BTC among urban cases, which continued to increase rapidly at older ages, while rural subjects had a slower pace of increase.

## Discussion

 In the recent study, we assessed the epidemiology of BTC in the Golestan province from 2004 to 2016. Overall, the ASRs of BTC were 1.7 in females and 1.4 among males. Nearly at the same time, the worldwide ASRs of BTC were 2.4 and 2.2 in females and males, respectively.^6^ The highest ASRs were reported in Chile, Japan, and South Korea, whereas the lowest ASRs were recorded in Iraq, Tajikistan, and Uganda.^14^ Asian countries are among the top high- and low-risk areas for BTC incidence. Considering the high ASR of BTC among central and East Asia, Europe, and other Western regions, it seems that Middle Eastern countries have a unique state regarding the BTC incidence^14^. This fact may result from the varying prevalence of risk factors such as gallbladder diseases, abnormal pancreaticobiliary duct junction, choledochal cysts, and biliary infection in these areas. For instance, the prevalence of gallstones as the most important risk factor of BTC is significantly lower in Iran compared to Caucasian populations in developed countries.^15,16^ In addition to the previous reports about the incidence of BTC in Iran,^7,8,17^ our findings indicate that the Golestan province could be classified as a low-risk area for BTC.

 At the global level, the incidence of BTC has increased over the past three decades, whereas both females and males had downward ASR.^14^ Our study showed that the incidence of BTC has increased within the study period in Golestan, but ASR trends were not in the same direction; females had a stable ASR, while males showed an increasing trend. Although the ASR of BTC in males decreased worldwide, increasing trends were also reported in other Asian countries, such as Georgia, Saudi Arabia, China, and Armenia.^14^ The ASR in the United States for both sexes exhibited a downward trend with more stability among males between 1999 to 2013.^18^ In different parts of Europe, such as France, Denmark, the United Kingdom, and Sweden, decreasing trends in ASR were reported for both females and males.^13,19-21^ Overall, these findings showed that the trend in the ASR of BTC had different patterns between Western and Eastern countries. The Asian population is more likely to have certain risk factors for BTC compared to other populations, including dietary choices, biliary infections and gallstones, and hepatitis B and C virus infection.^22-25^ These significant characteristics may explain the various patterns of ASR changes in this region of the world, including Iran.

 It is not clear why the BTC incidence decreased over the past years. In the literature, as gallstone disease is the major risk factor for BTC, the development of laparoscopic cholecystectomy surgery and its increased use were introduced as the main reasons for the downward incidence of BTC.^26,27^ However, the investigations showed that this declining trend had begun decades before the introduction of laparoscopic cholecystectomy; in addition, no temporal association was found between laparoscopic cholecystectomy rate and the incidence of gallbladder cancer.^28^ Another significant risk factor for gallbladder cancer is obesity. Although the obesity epidemic has continued in Western countries during the past decades and it is expected to see an increasing incidence in BTC, the findings show a different pattern. Determining the main reasons for this fact is still the subject of ongoing debates and needs to be investigated in the future.

 In contrast to Western nations, the Golestan province had a lower frequency of obesity and gallstone disease, but a greater incidence of hepatitis B virus infection in males.^29,30^ Additionally, the male population of Golestan use opium extensively.^31^ Different prevalence of the BTC risk factors alongside the BTC incidence and trend among males in the Golestan province suggested that a particular confluence of risk factors in this area predisposes men to BTC. Further investigation is needed in this province to draw a larger picture of BTC risk factors.

 The comparison of BTC incidence between rural and urban populations in this study showed that for both sexes, BTC occurred more frequently in urban areas. A few investigations have compared the BTC incidence in rural and urban areas; most reported that BTC incidence is higher in urban areas.^32,33^ The role of urbanization on BTC incidence has remained unclear. However, factors such as the prevalence of Western diet, obesity, less activity, and better access to medical services in urban areas could justify this fact.

 Like most other malignancies, the incidence of BTC increases with age. In terms of the age-specific incidence rate, in this study, the incidence of BTC in males began its upward slope from the age of 45, accelerated at the age of 70-74, and reached its peak in the age-group 85 years and older. Females also showed the same pattern but with a lower rate of acceleration in the age groups over 75. In the United States, the incidence rose significantly in the age-group 65‒74 and continued to reach the highest age-adjusted incidence rate among the age-group 85 and older.^18^ Also, In Sweden, the same statics were recorded, and the age-group over 80 had the greatest proportion of case incidence.^20^ The global burden of disease study (GBD) reported that the highest incidence of BTC occurred in the age-group 75‒79 worldwide.^14^ All these findings are consistent with the global rate and show that people at the age of 65 years and older are at higher risk of BTC.

 There were some limitations in the present study that did not affect the quality of results but could be taken into account for improving future studies. First, the definite diagnosis of cancer type and reporting the related details were still limited in GPCR. Second, lack of information about the mortality rates and ratios restricted us in evaluating the outcomes of BTC in Golestan. Since GPCR is at the beginning of its way to becoming a mature cancer registry, the mentioned limitations will be solved soon.

## Conclusion

 In conclusion, these findings together provide a significant public health message. We found a higher incidence of BTC in the demographic group with the combination of three factors: male gender, age over 65, and urban residence. As BTCs are lethal malignancies and effective preventive interventions are available such as laparoscopic cholecystostomy, identification of high-risk patients can lead to better management and saving more lives.
